# Comment on Dorobisz et al. Assessment of Prognostic Factors, Clinical Features Including the Microbiome, and Treatment Outcomes in Patients with Cancer of Unknown Primary Site. *Cancers* 2024, *16*, 3416

**DOI:** 10.3390/cancers16233911

**Published:** 2024-11-22

**Authors:** Tatsuki Itagaki, Machiko Kasai, Ken-ichiro Sakata, Akira Hasebe

**Affiliations:** 1 Oral Diagnosis and Medicine, Faculty of Dental Medicine, Graduate School of Dental Medicine, Hokkaido University, Kita-13 Nishi-7, Kita-ku, Sapporo 060-8586, Japan; sakata-0303@den.hokudai.ac.jp; 2 Microbiology, Faculty of Dental Medicine, Graduate School of Dental Medicine, Hokkaido University, Kita-13 Nishi-7, Kita-ku, Sapporo 060-8586, Japan; mtakagi@den.hokudai.ac.jp (M.K.); akkun@den.hokudai.ac.jp (A.H.)

Dorobisz et al. published an interesting paper entitled “Assessment of Prognostic Factors, Clinical Features Including the Microbiome, and Treatment Outcomes in Patients with Cancer of Unknown Primary Site” [1]. We would like to present our comment from the perspective of bacteria researchers. The influence of the microbiome has been attracting attention in recent years [1]. Dorobisz et al.’s viewpoint is excellent, but their analysis and interpretation leave room for improvement. Many researchers have reported that Fusobacterium and Porphyromonas are often detected in the presence of cancer, and Streptococcus and Lactobacillus are common bacteria living in the oral cavity and are suspected to be related to inflammation and oral cancer.

Dorobisz et al. considered the following: “In the discussed study, patients with Cancer of unknown primary site (CUP) had a reduced amount of Streptococcus and Lactobacillus in the oral microbiome compared to the control group. Reduced amounts of Streptococcus was also associated with an increased risk of Radiation-induced oral mucositis (RIOM) and shortened survival rate in patients with CUP”. Their microbiome data only show percentages; their results may be a result of compositional data. In other words, due to the compositional data’s nature, an increase in a specific component will result in a decrease in another [2]. Moreover, odds ratios are measures of association and are used for count data, but Dorobisz et al. used the index for compositional data [1]. Since Culture Results are count data, logistic regression analysis is possible. However, logistic regression analysis cannot be applied to microbiome analysis, which is a compositional data analysis [2]. The ratio of the lower limit to the upper limit of the 95% confidence interval is sometimes used as an index of the reliability of the odds ratio: 0.5 or more is acceptable, 0.25 or more is moderate, and less than 0.25 is unreliable. Considering the characteristics of the data and results presented by Dorobisz et al., we recommend combining several methods, such as correcting the total bacterial count using Culture Results and performing bacterial flora analysis. This will free us from the use of percentage data and reveal the truth.

We generally agree with Dorobisz et al. However, Dorobisz et al. have conducted much research, so we would like them to provide us with the results and their interpretations in a better way. In this way, they can achieve results that cannot be obtained through bacterial flora analysis alone, and we will uncover the truth.
